# Dynamic contrast-enhanced MRI shows altered blood–brain barrier function of deep gray matter structures in neuroborreliosis: a case–control study

**DOI:** 10.1186/s41747-023-00365-6

**Published:** 2023-09-15

**Authors:** Elisabeth S. Lindland, Anne Marit Solheim, Silje Andreassen, Robin Bugge, Randi Eikeland, Harald Reiso, Åslaug R. Lorentzen, Hanne F. Harbo, Mona K. Beyer, Atle Bjørnerud

**Affiliations:** 1 Department of Radiology, Sorlandet Hospital, Sykehusveien 1, N-4809 Arendal, Norway; 2 Division of Radiology and Nuclear Medicine, Oslo University Hospital, Oslo, Norway; 3 Institute of Clinical Medicine, University of Oslo, Oslo, Norway; 4 Department of Neurology, Sorlandet Hospital, Kristiansand, Norway; 5 Institute of Clinical Medicine, University of Bergen, Bergen, Norway; 6 Department of Pediatrics, Sorlandet Hospital, Arendal, Norway; 7 Department of Physics and Computational Radiology, Oslo University Hospital, Oslo, Norway; 8 The Norwegian National Advisory Unit On Tick-Borne Diseases, Sorlandet Hospital, Kristiansand, Norway; 9 Faculty of Health and Sport Sciences, University of Agder, Kristiansand, Norway; 10 Department of Neurology, Oslo University Hospital, Oslo, Norway; 11 Department of Physics, University of Oslo, Oslo, Norway

**Keywords:** Blood–brain barrier, Encephalitis (tick-borne), Gray matter, Lyme neuroborreliosis, Magnetic resonance imaging

## Abstract

**Background:**

Main aim was assessment of regional blood–brain barrier (BBB) function by dynamic contrast-enhanced magnetic resonance imaging (DCE-MRI) in patients with neuroborreliosis. Secondary aim was to study the correlation of BBB function with biochemical, clinical, and cognitive parameters.

**Methods:**

Regional ethical committee approved this prospective single-center case–control study. Within 1 month after diagnosis of neuroborreliosis, 55 patients underwent DCE-MRI. The patient group consisted of 25 males and 30 females with mean age 58 years, and the controls were 8 males and 7 females with mean age 57 years. Pharmacokinetic compartment modelling with Patlak fit was applied, providing estimates for capillary leakage rate and blood volume fraction. Nine anatomical brain regions were sampled with auto-generated binary masks. Fatigue, severity of clinical symptoms and findings, and cognitive function were assessed in the acute phase and 6 months after treatment.

**Results:**

Leakage rates and blood volume fractions were lower in patients compared to controls in the thalamus (*p* = 0.027 and *p* = 0.018, respectively), caudate nucleus (*p* = 0.009 for both), and hippocampus (*p* = 0.054 and *p* = 0.009). No correlation of leakage rates with fatigue, clinical disease severity or cognitive function was found.

**Conclusions:**

In neuroborreliosis, leakage rate and blood volume fraction in the thalamus, caudate nucleus, and hippocampus were lower in patients compared to controls. DCE-MRI provided new insight to pathophysiology of neuroborreliosis, and can serve as biomarker of BBB function and regulatory mechanisms of the neurovascular unit in infection and inflammation.

**Relevance statement:**

DCE-MRI provided new insight to pathophysiology of neuroborreliosis, and can serve as biomarker of blood–brain barrier function and regulatory mechanisms of the neurovascular unit in infection and inflammation.

**Key points:**

• Neuroborreliosis is an infection with disturbed BBB function.

• Microvessel leakage can be studied with DCE-MRI.

• Prospective case–control study showed altered microvessel properties in thalamus, caudate, and hippocampus.

**Graphical Abstract:**

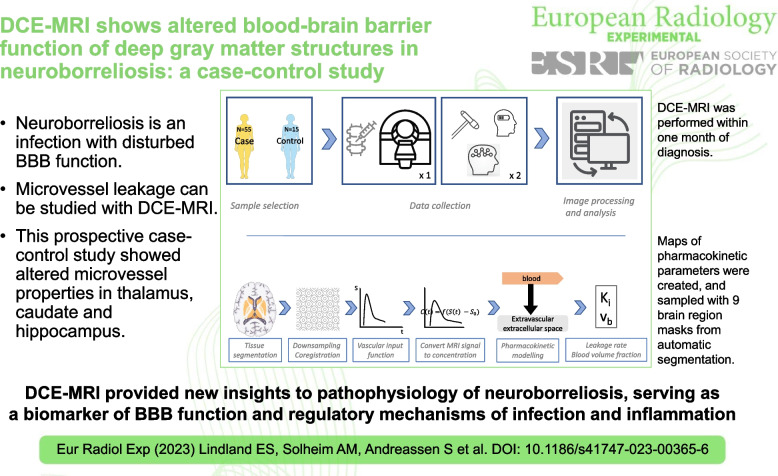

**Supplementary Information:**

The online version contains supplementary material available at 10.1186/s41747-023-00365-6.

## Background

Neuroborreliosis is a tick-borne spirochetal infection where increase of protein, albumin, and white blood cells in the cerebrospinal fluid (CSF) is higher than in viral meningitis, indicating considerable nervous system inflammation and disturbed integrity of blood–brain barrier (BBB) [1]. The BBB regulates movement of ions, molecules, and cells between the blood and brain [2], and even subtle changes may alter functions of the neurovascular unit and cause injury to the central nervous system [3]. Studies of residual complaints after treatment of neuroborreliosis point towards an association with treatment delay more than the CSF markers [4, 5]. However, CSF markers cannot provide information on function in selective brain regions, and therefore lack accuracy. Methods to make regional measurements in the brain have a major potential to provide new insight to the pathophysiology of neuroborreliosis, and to serve as biomarker in studies of treatment response and outcome.

The signal intensity-to-time curve from dynamic contrast-enhanced (DCE) magnetic resonance imaging (MRI) together with application of pharmacokinetic models provides estimates for microvessel properties of the tissue, and is considered an imaging biomarker for BBB function [6]. In tissues with subtle capillary permeability, the kinetic parameters estimated are the leakage rate of tracer from blood to tissue, and the fraction of blood volume in the tissue. Several research groups have demonstrated use of this technique for studying ageing and brain diseases such as small vessel disease, dementia, and multiple sclerosis [6–13], but equivalent studies in patients with infection are lacking. Non-specific complaints, such as fatigue and reduced cognitive function, can be encountered after infection both inside and outside of the nervous system [14–16], and the study of regional brain microvessel properties in association with such complaints may entail valuable hypotheses regarding the pathophysiology of these complaints.

The study presented here provides a baseline investigation of DCE-MRI acquired in patients with neuroborreliosis compared to healthy control subjects. The study aim was to test the following two hypotheses: (1) leakage rate and blood volume fraction in selected brain regions are different in patients compared to healthy controls; and (2) leakage rate in the selected brain regions correlates with CSF albumin, protein and pleocytosis, clinical disease severity, cognitive function, or fatigue.

## Methods

**Fig. 1:**
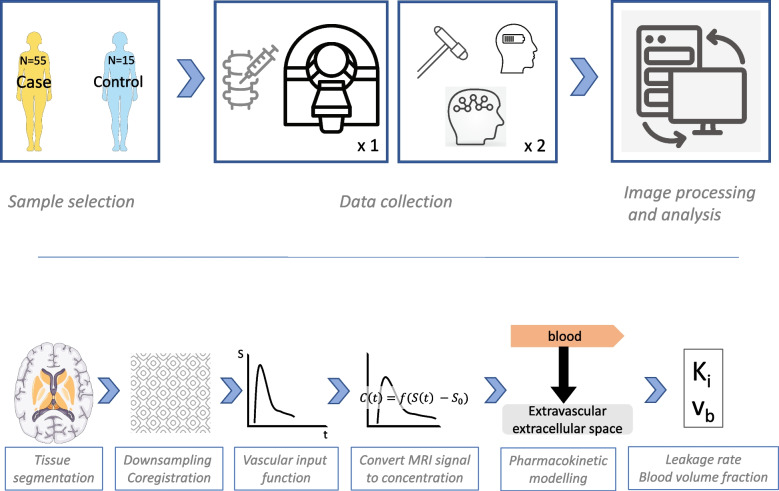
Graphical overview of the study method. In this study, 55 neuroborreliosis cases came to brain MRI within 1 month of diagnosis, and 15 age- and sex-matched controls were recruited. For the cases only, clinical, cognitive, and fatigue severity data were collected at the time of inclusion and 6 months after treatment. Image post processing and analysis included automatic gray-white matter tissue segmentation, downsampling, and coregistration of the three-dimensional T1-weighted sequence to the dynamic contrast-enhanced MRI sequence, manual identification of vascular input function per subject, conversion of MRI signal intensity to concentration using individual baseline T1 measurement, and pharmacokinetic modelling with Patlak equation fit which provides estimates for microvascular leakage rate and blood volume fraction. *C* Concentration, *f* Function, *K*_*i*_ Leakage rate, *MRI* Magnetic resonance imaging, *S* Signal intensity, *t* Time, *v*_*b*_ Blood volume fraction

A graphical overview of the study is provided in Fig. 1.

### Subjects

The regional ethics committee and institutional data protection services approved of this case–control study, and participants gave written informed consent. This is a substudy in the BorrSci research project, which includes a randomized clinical trial and an MRI-neuropsychology prospective longitudinal study [17–21]. The patients were included between December 1, 2015 and December 18, 2018. Inclusion criterion was diagnosis of definite or possible neuroborreliosis according to the European Federation of Neurological Societies’ diagnostic guidelines [22]. The guidelines state that all three of the following criteria should be fulfilled for diagnosis of definite, and two of them for possible neuroborreliosis: Neurological symptoms without other obvious cause, CSF pleocytosis and intrathecal production of *Borrelia Burgdorferi* specific antibodies. Individuals scheduled for contrast-enhanced MRI due to routine follow-up of lesions in the head and neck region were invited to participate as control subjects. They were included from September 25th, 2018 to May 2nd, 2019. Exclusion criteria were age less than 18 years, pregnancy, breast-feeding, claustrophobia, implants with safety issues, and contraindications for contrast agent. Also, fourteen subjects underwent DCE scanning without contrast agent to provide data for signal drift.

### Biochemical, clinical, and cognitive function data

Patients had CSF analysis prior to MRI, controls did not. The biochemical markers used for inflammation and BBB leakage were CSF cell count, CSF total protein and CSF-serum albumin ratio. We registered duration of neurological symptoms and time interval between CSF sample collection and MRI. MRI and CSF data were only collected in the early phase, clinical and cognitive data were collected from the patients in both the early phase and 6 months after treatment. Degree of clinical symptoms and objective clinical findings was summarized in a clinical composite score, which is described in the clinical trial report [21]. Fatigue was assessed with Fatigue Severity Scale [23]. In the early phase, short-term and working memory function was assessed with digit span forward and backward test. Six months after treatment, verbal memory test score was used. The cognitive tests were selected based on previous findings related to executive and memory functions [24]. Hematocrit was assessed for both groups to avoid bias.

### Imaging

All participants were scanned on the same 3.0-T MRI, Magnetom Skyra (Siemens Healthineers, Erlangen, Germany), within 1 month after diagnosis. The anatomical sequence was a sagittal Three-dimensional (3D) T1-weighted magnetization-prepared rapid gradient-echo, MPRAGE. The DCE sequence was a 3D T1-weighted volumetric interpolated breath-hold examination, VIBE, spoiled gradient echo with 80 frames and temporal resolution 7.2 s. Contrast agent was injected intravenously after 10 frames (Dotarem, Guerbet, Villepinte, France) 0.2 mL/kg, 0.5 mmol/mL, at the rate of 2 mL/s, followed by 20 mL of saline solution (power injector, MedRad Spectris Solaris EP, Bayer, Leverkusen, Germany). Precontrast T1 mapping was done with a dual flip angle 3D spoiled gradient-echo sequence with geometry identical to that of the DCE sequence. Detailed scan parameters are presented in Supplemental Table S1.

### Image analysis

Voxel-based maps of pharmacokinetic parameters were created, and these were sampled with auto-generated tissue masks. Automatic segmentation of 3D T1-weighted acquisition was done with FastSurfer [25]. The segmentation results were visually checked by a neuroradiologist (E.S.L.). The regions selected for analysis were: frontal, parietal and temporal cortex, thalamus, caudate, putamen, hippocampus, brain stem and cerebellar white matter. These regions were chosen to cover areas that we have experienced to be involved in encephalitis due to neuroborreliosis (thalamus, brain stem, and white matter of cerebellum), reports of more reduction in executive functions (frontal region), and single-photon emission computed tomography and positron emission computed tomography studies with finding of lower activity (temporal, parietal, and limbic regions), as well as age-related increase in leakage (hippocampus) [9, 24, 26, 27]. Supratentorial white matter commonly has hyperintense changes in the middle-aged and elderly, and this segment was not included due to variable leakage changes in abnormal white matter [28].

The DCE data was corrected for in-plane motion (translation, rotation). Noise level was automatically determined in each dataset, setting the threshold for pixels to include in analysis. Coregistration of anatomical and functional sequences was done by creating a transformation matrix between averaged pre-contrast DCE image volume and the 3D T1-weighted acquisition. The same transformation matrix was applied to the T1 map. Downsampling of tissue masks was done to fit the spatial resolution of the DCE acquisition. Coregistration and downsampling were performed in SPM12 (https://www.fil.ion.ucl.ac.uk/spm/) running in MATLAB R2019b (MathWorks, Natick, MA, USA).

DCE data was fitted to the Patlak model, using Levenberg–Marquardt non-linear least squares algorithm [29]. This model assumes unlimited blood flow and uni-directional flux of contrast agent from the intravascular to the extravascular-extracellular space and provides two estimates of microvessel properties: (1) the rate of leakage (K_i_) of tracer from blood to the extravascular-extracellular compartment; and (2) the volume fraction of capillary blood in tissue (v_b_). The Patlak model equation is

$${\mathrm{C}}_{\mathrm{t}}\left(\mathrm{t}\right)={\mathrm{K}}_{\mathrm{i}}{\int }_{0}^{\mathrm{t}}{\mathrm{C}}_{\mathrm{b}}\left(\uptau \right)\mathrm{d\tau }+{\mathrm{v}}_{\mathrm{b}}{\mathrm{C}}_{\mathrm{b}}\left(\mathrm{t}\right)$$

where C_t_(t) and C_b_ are the contrast agent concentration in tissue and blood, respectively. DCE signal intensity was converted to contrast agent concentration, using pixel-wise baseline T1-values obtained from the dual flip angle scan [30].

The pharmacokinetic modelling approach may take into account that the tracer is only distributed in the plasma volume and not the whole blood volume, and the hematocrit value is used for this correction, yielding transfer constant (K^trans^) and plasma volume fraction (v_p_) instead of leakage rate (K_i_) and volume fraction of capillary blood in tissue (v_b_).

$$K_i(1-hct)=K^{trans}\;and\;v_b(1-hct)=v_p$$

Individual values for hematocrit level were not available from blood tests taken on the same day as the MRI examination, hence we estimated full blood values, K_i_ and v_b_. Hematocrit level was compared between the groups to avoid bias from difference in hematological status. Hematocrit or hemoglobin measurement from within 1 month prior for the patient group and within 6 months prior for the control group was used. Only eight subjects had a hematocrit measurement. A widely used estimate for hematocrit from hemoglobin values was applied for the remaining subjects, the equation Hct % = Hb (g/dL) × 3 [31].

Accuracy of parameter estimation in DCE-MRI is dependent on correct identification of vascular input function (VIF) [32]. Use of population VIFs has previously been shown to offer improved reproducibility in DCE parameter estimates [33]. Given the challenge of robust identification of VIF, analysis was performed both with individually determined VIFs and using a population VIF constructed from the average of the individual VIFs. The VIFs were identified manually by the same neuroradiologist (E.S.L.) for each subject from the DCE series, both from a large intracranial artery and dural venous sinus. A small circular ROI was placed in the transverse or sigmoid sinus and in the internal carotid artery at the level of the cavernous sinus, attempting a size and placement that provided highest possible peak, sharp rise and avoiding a double peak or signal drop from inflow artifact. Any temporal shift between the VIF and tissue response curve was automatically pixel-wise corrected by repeating the full kinetic analysis multiple times and temporally displacing the VIF by one timepoint (total ± 2 timepoints) between each analysis and then selecting the VIF giving the lowest residual error in the curve fit. Consensus recommendation suggests venous input function for analysis in tissues with low range leakage rates [34]. This reduces partial volume and in-flow effects compared to an arterial input function. Contrast agent concentration-to-time plots for the individual and population-averaged venous vascular input functions are shown in Supplemental Figure S1. Results from the kinetic analysis with population-averaged venous input function are presented here, while results from individual venous input function and arterial input functions, as well as assessment of agreement between kinetic parameters obtained with the different VIF methods, are presented as Supplementary material (Tables S4–10). Also, a model-free approach with area under the curve analysis, and assessment of signal drift, are provided in Supplementary material (Table S3 and Figure S1).

Kinetic analysis and T1 mapping were performed using NordicICE version 4 (NordicImagingLab, Bergen, Norway). For each tissue mask, voxels with parameter value negative or zero were excluded. Mean values per mask for all parametric maps were used for group comparison, correlation and reliability analysis.

### Statistical analysis

Statistical analysis was performed using SPSS version 28 (IBM, Armonk, NY, USA). Mann–Whitney *U* test was used to test difference of model-free and kinetic parameters between the groups per tissue mask, and Spearman correlation coefficient was used for explorative analyses to study association of leakage rate with the secondary endpoints, the CSF, clinical and cognitive function markers. *χ*^2^ test was used to compare sex distribution between the groups. Student’s *t* test was used to compare age, hematocrit level, volumes of tissues masks and head motion. Agreement of kinetic parameter estimates with various VIFs was evaluated with Cronbach alpha and intraclass correlation coefficient. A *p* value lower than 0.05 was chosen as the significance level. Bonferroni correction was used due to multiple comparisons, the raw *p* values were multiplied by the number of brain regions that were tested.

## Results

**Fig. 2:**
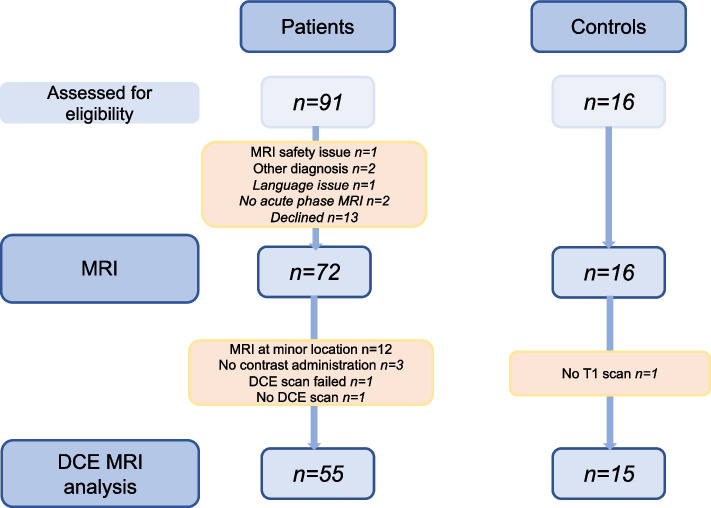
Flow chart of subject enrollment. To avoid technical bias, only subjects scanned at one location were used in this dynamic contrast enhanced substudy

The patient group consisted of 25 males and 30 females aged 58 ± 13 years (mean ± standard deviation). The control group were 8 males and 7 females aged 57 ± 17 years. A flow-chart of subject enrollment and exclusion is provided in Fig. 2. There was no significant difference in sex distribution between the groups (*χ*^2^ = 0.063, *p* = 0.803, φ = 0.065), no difference in age (mean difference 1.1 years, *p* = 0.791), no difference in hematocrit level (mean difference − 0.01, *p* = 0.368), no difference in head motion (translation, mean difference = 0.001, *p* = 0.920; rotation, mean difference = 0.005, *p* = 0.730), and no differences in volumes of autogenerated tissue masks (Supplemental Table S2). Diagnosis of neuroborreliosis was definite in 45 of 55 subjects (81.8%) Further clinical and laboratory data are provided in Table 1.

**Table 1:** Clinical and laboratory characteristics of patients and controls

	**Patients (*n* = 55)**	**Controls (*n* = 15)**	***p* value**
Sex, males/females	25/30	8/7	0.803
Age, years (mean ± SD; range)	58 ± 13; 21–82	57 ± 17; 21–81	0.791
Hematocrit level	0.41 ± 0.04; 0.34–0.51	0.43 ± 0.03; 0.35–0.48	0.368
Duration neurological symptoms, days	27 ± 29; 2–180		
Interval CSF test-MRI, days	16 ± 8; 2–31		
CSF cells/mm^3^	190 ± 166; 7–752		
CSF protein, g/L	1.34 ± 0.79; 0.32–3.86		
CSF-serum albumin ratio (× 0,001)	22.3 ± 13.8; 5.2–68.4		
*Bb* antibody index positive (*N*)	45		

For continuous variables, data are given as mean ± standard deviation and range. Eight patients had negative *Bb* antibody index and their mean duration of symptoms was 12 days (range 4–28). Antibody index was missing for two patients. *Bb Borrelia Burgdorferi*, *CSF* Cerebrospinal fluid

**Fig. 3:**
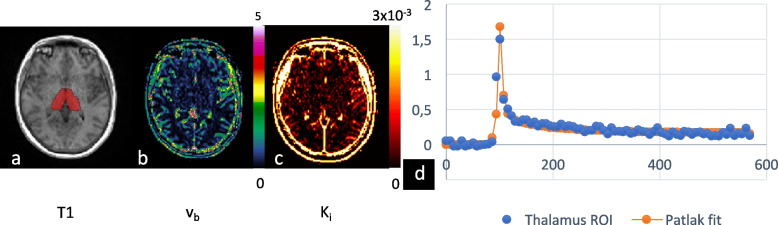
Output of tissue segmentation and post-processing. Anatomic image (**a**), kinetic parameter maps (**b**, **c**) and concentration-to-time curve (**d**) from a study participant: T1-weighted magnetization-prepared rapid gradient-echo, MPRAGE, image with autogenerated tissue mask of thalamus marked as red voxels (**a**), v_b_ (**b**), and K_i_ (**c**) maps. Blue plots in the graph (**d**) show contrast agent concentration (*y* axis) in thalamus measured over time (*x*-axis). The Patlak fitted curve is drawn in red, and indicates a good fit. *K*_*i*_ leakage rate, *v*_*b*_ blood volume fraction

Example of anatomical tissue mask, v_b_ and K_i_ maps at the level of thalamus together with concentration-to-time plot with fitted Patlak curve in a study subject is provided in Fig. 3. Values for area under the curve per region with group comparison are provided in Supplemental Table S3.

**Fig. 4:**
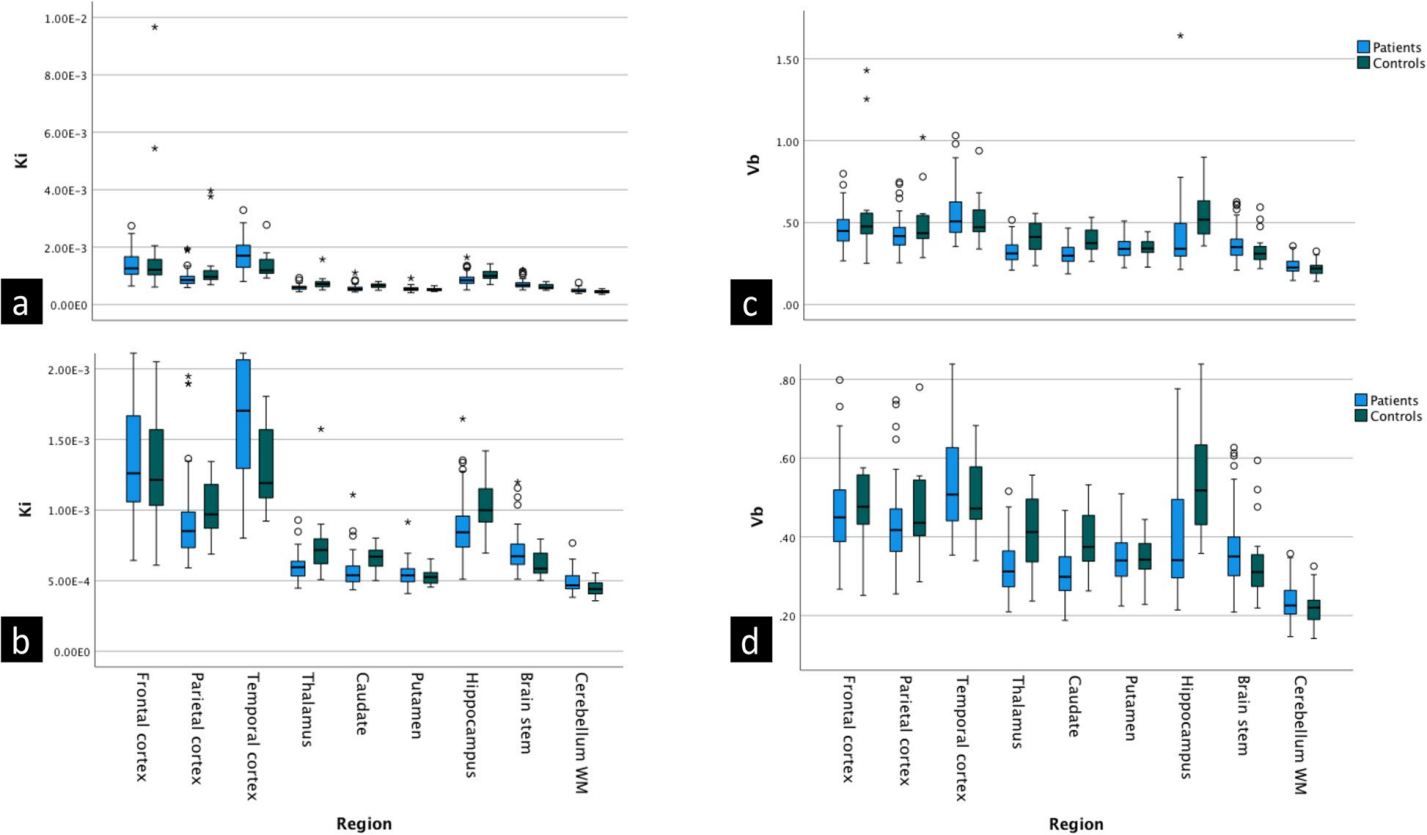
Group comparison of kinetic parameters. Box plots for leakage rates (**a**, **b**) and blood volume fractions (**c**, **d**), plots in the upper row (**a**, **c**) include the extreme outliers, and the *y*-axis scale is adjusted in the lower row plots (**b**, **d**) to display the boxes more clearly. The extreme outliers were segments with a large difference in mean and median values of kinetic parameters, and are likely due to partial inclusion of some larger vessels. We did not exclude them from analyses. *K*_*i*_ Leakage rate, *v*_*b*_ Blood volume fraction

**Fig. 5:**
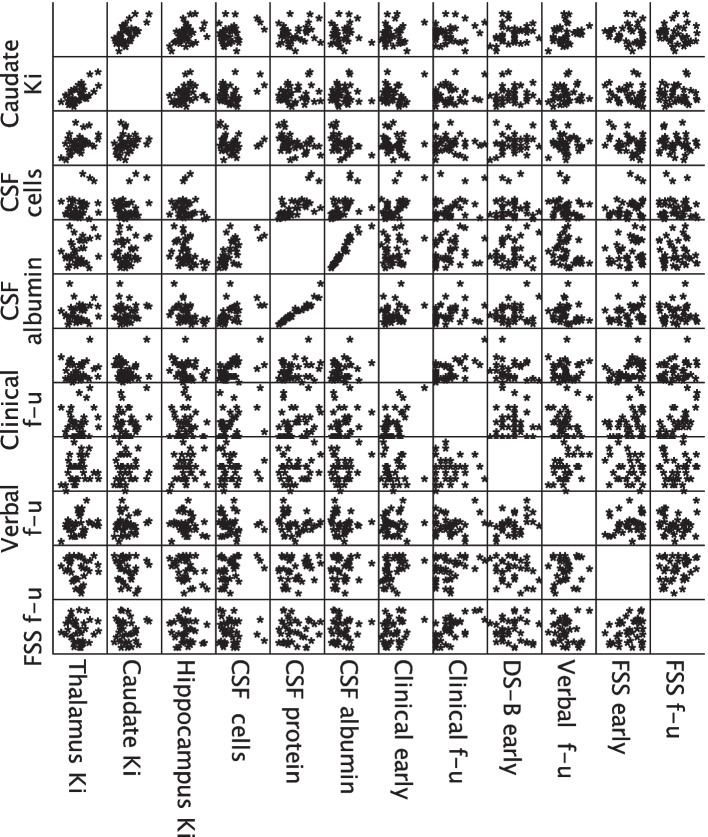
Correlation analysis between leakage rate and fatigue, biochemical, clinical and cognitive parameters. Scatter matrix with selected parameters from the correlation analyses, a full list of correlation coefficients and *p* values is provided in Supplemental Table S11. There was moderate negative correlation between leakage rate in hippocampus and cerebrospinal fluid total protein. There were no other significant correlations. *Clinical early* Clinical composite score at inclusion, *CSF* Cerebrospinal fluid, *DS-B* Digit span backward test score, *FSS* Fatigue Severity Scale, *f-u* Follow-up, *K*_*i*_ Leakage rate, *Verbal* Verbal memory test score

Leakage rate and blood volume fraction were lower in patients compared to controls in thalamus, caudate nucleus and hippocampus. The significance of difference in leakage rate for hippocampus only marginally did not survive the Bonferroni correction (*p* = 0.054). Moreover, leakage rates were higher in the brainstem, cerebellum white matter, and temporal cortex of patients, but none of these survived the Bonferroni correction (*p* values from 0.090 to 0.207). Values for the kinetic parameters per region are shown with box plots in Fig. 4. Results of group comparison are provided in Tables 2 and 3. Comparable results for kinetic parameter estimates were obtained using the other VIFs (Supplemental Tables S4 − S9, agreement statistics provided in Supplemental Table S10). There was a significant moderate degree of negative correlation between the leakage rate in the hippocampus and CSF protein (ρ =  -0.39, adjusted *p* value = 0.036). No association was found for the other regions, and no association was found between the leakage rates and clinical severity, cognitive tests or fatigue. A scatter matrix is provided in Fig. 5, while a complete list of correlation coefficients and *p* values are provided in Supplemental Table S11.

**Table 2:** Leakage rates and group comparison

K_i_ (× 10^−3^/min)	Patient group mean (median, SD)	Control group mean (median, SD)	Mean difference (95% CI)	Mann–Whitney *U* test *p* value
Frontal cortex	1.38 (1.26, 0.49)	2.05 (1.21, 2.40)	-0.67 (-2.00–0.67)	0.824
Parietal cortex	0.94 (0.85, 0.30)	1.35 (0.97, 1.02)	-0.42 (-0.99–0.16)	0.052
Temporal cortex	1.70 (1.70, 0.51)	1.38 (1.19, 0.48)	0.32 (0.03–0.61)	0.010* (0.090)
Thalamus	0.60 (0.60, 0.09)	0.75 (0.72, 0.25)	-0.15 (-0.30–0.01)	0.003* (0.027*)
Caudate	0.57 (0.54, 0.11)	0.66 (0.67, 0.09)	-0.09 (-0.28 to -0.03)	0.001* (0.009*)
Putamen	0.55 (0.54, 0.08)	0.53 (0.53, 0.06)	0.02 (-0.03–0.06)	0.479
Hippocampus	0.88 (0.84, 0.22)	1.04 (1.00, 0.19)	-0.16 (-0.28 to -0.03)	0.006* (0.054)
Brainstem	0.71 (0.67, 0.15)	0.62 (0.58, 0.09)	0.09 (0.01–0.17)	0.022* (0.198)
Cerebellum white matter	0.49 (0.47, 0.07)	0.45 (0.44, 0.06)	0.05 (0.00–0.09)	0.023* (0.207)

Leakage rate, K_i_, was generated with Patlak model and population averaged vascular input function from venous sinus. Mann–Whitney *U* tests indicated lower leakage rate in thalamus, caudate and hippocampus of patients compared to healthy controls (*p* values in parentheses are after Bonferroni correction)

^*^denotes significance. *CI* Confidence interval, *SD* Standard deviation

**Table 3:** Blood volume fractions and group comparison

Blood volume fraction (v_b_)	Patient group mean (median, SD)	Control group mean (median, SD)	Mean difference (95% CI)	Mann–Whitney *U* test *p* value
Frontal cortex	0.46 (0.45, 0.12)	0.57 (0.48, 0.33)	-0.11 (-0.29 to -0.01)	0.274
Parietal cortex	0.43 (0.42, 0.11)	0.50 (0.44, 0.18)	-0.07 (-0.14–0.01)	0.210
Temporal cortex	0.55 (0.51, 0.15)	0.53 (0.47, 0.15)	0.02 (-0.07–0.10)	0.543
Thalamus	0.32 (0.31, 0.07)	0.41 (0.41, 0.10)	-0.09 (-0.15 to -0.03)	0.002* (0.018*)
Caudate	0.31 (0.30, 0.06)	0.40 (0.37, 0.08)	-0.09 (-0.13 to -0.05)	0.001*(0.009*)
Putamen	0.34 (0.34, 0.06)	0.34 (0.34, 0.06)	-0.004 (-0.04–0.03)	0.791
Hippocampus	0.41 (0.34, 0.21)	0.56 (0.52, 0.21)	-0.14 (-0.26 to -0.03)	0.001*(0.009*)
Brainstem	0.36 (0.35, 0.10)	0.34 (0.31, 0.11)	0.02 (-0.04–0.08)	0.176
Cerebellum white matter	0.23 (0.23, 0.05)	0.22 (0.22, 0.05)	0.01 (-0.02–0.04)	0.444

The blood volume per unit volume of tissue (blood volume fraction), v_b_, was generated with Patlak model and population averaged vascular input function from venous sinus. Mann–Whitney *U* tests indicated significantly lower blood volume fractions in thalamus, caudate, and hippocampus of the patients (*p* values in parentheses are after Bonferroni correction). *CI* Confidence interval, *SD* Standard deviation

Result of signal drift assessment is provided in Supplementary Figure S2.

## Discussion

This study used DCE-MRI and pharmacokinetic compartment modelling to generate estimates for microvessel properties. Leakage rates and blood volume fractions were decreased in thalamus, caudate nucleus and hippocampus of neuroborreliosis patients compared to controls. For the hippocampus, this was negatively correlated with CSF BBB leakage markers. There was no association with clinical disease severity, fatigue, or cognitive function in early disease phase or at follow-up. Decreased leakage rate is a surprising finding, as most studies report increased values in disease and ageing [6, 7, 9, 12, 13], and increased markers for BBB leakage is often pronounced in neuroborreliosis [1]. Among reported findings are increased leakage in hippocampus associated with ageing and mild cognitive impairment [9], and in white matter and thalamus of patients with multiple sclerosis which was predicted by disease activity [7]. Few studies reported results for blood or plasma volume fraction, but a study of patients with early Alzheimer disease found similarly to our results reduced plasma volume in gray matter, but no significant difference in leakage rate [11].

Capillary permeability assessment by DCE-MRI is an emerging technique where complex image processing limits comparability of studies [34]. Also, it is not possible from the model to state if the observed reduced leakage rate is from lower microvessel permeability per se. This is because lower blood volume fraction is associated with lower sum of vessel surface area and therefore reduced leakage rate despite equal or even higher permeability of the endothelium per unit of vessel surface area [34], and this underscores the importance that studies should report all parameters from the pharmacokinetic modelling procedure.

Our findings of a significant reduction in both leakage and volume parameters in important subcortical gray matter structures, may be attributed to pathophysiologic changes specific for neuroborreliosis, or to regional variations of autoregulatory mechanisms of the neurovascular unit. Primary targets for this regulation would be vascular smooth muscle cells, pericytes and astrocyte endfeet, and such regulation could serve to minimize brain damage. Also, we observed a tendency for increased leakage rate with no difference in blood volume fraction for brainstem, cerebellar white matter and temporal cortex (significant difference of leakage rates only prior to Bonferroni correction) and this could imply that protective vascular autoregulation is more developed for deep gray matter compared to white matter and cortical areas. This could also explain the difference from observations of increased leakage rates in studies of more chronic inflammatory or degenerative diseases such as multiple sclerosis and dementia [7, 9], where such regulatory mechanisms may no longer be intact. Additionally, the suggestion of autoregulation is also supported by the finding of gray matter reduced plasma volume with no increase in leakage rate in early Alzheimer disease [11].

The level of protein and albumin in CSF are markers for BBB function, and CSF-serum albumin ratio is considered the superior test for leakage [35]. A minority of previous clinical studies have included biochemical BBB leakage parameter in their data collection, with findings of correlation between CSF-albumin ratio and leakage rate in abnormal white matter areas and hippocampus in cognitive impairment [9, 36]. They did not report an association for other regions. In our study of neuroborreliosis patients, total protein level in CSF showed moderate negative correlation to leakage rate of hippocampus. Correlation between CSF-serum albumin ratio and hippocampus leakage rate was only significant prior to Bonferroni correction. No correlation was found for the other brain regions. Although we only found a mild-moderate degree of correlation in the hippocampus, there could be an autoregulatory reduction in leakage rate as discussed in the previous section, and it seems to be associated with markers that indicate degree of inflammation and BBB breakdown.

The BBB is a multifaceted functional and anatomical entity. It is mainly comprised of endothelial cell properties such as proteins of the cell junctions, transporters, luminal glycocalyx and abluminal basal membrane as well as vascular smooth muscle cells, pericytes and astrocyte endfeet [2]. Therefore, studying the association of leakage rate of gadolinium based contrast agent with protein and albumin level in CSF, may be somewhat artificial. Among important differences is considerably smaller size of the contrast agent. Also, the association of lumbar CSF space to extravascular-extracellular compartment of brain tissue may be limited, as there are fundamental differences in the endothelial cell properties of brain tissue and choroid plexus where most of the CSF is generated [2].

Our patient group is highly representative for neuroborreliosis patients. European diagnostic criteria were applied [22], and patients treated inside and outside of hospital were included. Diagnosis was definite in 82% of patients, this proportion being in line with other studies [1, 37], which further ensures clinical generalizability. The possible cases are often due to early disease phase before *Borrelia burgdorferi* specific antibodies can be detected in the CSF and sensitivity of the antibody index can be low [38]. Still, the chance of a small proportion of cases without neuroborreliosis is a minor limitation.

Healthy controls provided reference values and some compensation for the limitations in the method that bias the kinetic parameter estimates, such as artifact level and noise. We addressed several factors that influence accuracy of the estimates. First, individual baseline T1 mapping was applied, and this increased the accuracy of contrast agent-induced T1 change quantification required for kinetic modelling. Second, Patlak model was used to estimate leakage rate and blood volume fraction as recommended for low permeability conditions [13, 34]. Also, different VIF approaches were studied with consistency of the main results, and signal drift was assessed.

DCE-MRI acquisition time of 9.6 min may be a limitation as it was relatively short compared to the aforementioned consensus paper recommendation of at least 15 min duration for studying conditions with low leakage rates [34]. A challenge of extending the acquisition time is increasing motion artefacts. A minor limitation to generalizability is that results may differ between the areas where ticks reside due to different bacteria species. We did not adjust for white matter lesion load, a potential for bias from leakage changes related to small vessel disease [39], but we have previously reported that Fazekas scores in this patient group do not differ from those of healthy controls [18]. The Bonferroni correction is a straightforward, but rather strict method to compensate for multiple comparisons. Choice of a different method may have provided statistically significant group differences in more brain regions in this study. Follow-up scanning was not performed and lack of longitudinal DCE-MRI data limits the study conclusion.

In conclusion, brain microvessel properties are altered in neuroborreliosis. We postulate that it may be attributed to autoregulatory mechanisms of the neurovascular unit that serve to minimize brain damage in an ongoing infection, and that such mechanisms may not be preserved in more chronic inflammatory or degenerative diseases. DCE-MRI is an important imaging method for studying the role of microvessel properties in pathophysiology and treatment response in infection and inflammation of the nervous system.

### Supplementary Information

**Additional file 1: Supplemental Table S1.** Scan parameters for the imaging sequences in the study. **Supplemental Table S2.** Volumes (mean (standard deviation)) of the selected anatomical tissue masks that were generated by automatic segmentation of the 3D T1 acquisition, and *p*-values from t-tests for group comparison. **Supplemental Table S3.** Area under the curve of the signal intensity-to-time plot per region, and *p*-values from Mann–Whitney U tests for group comparison. **Supplemental Figure S1** shows plots of all the individual venous VIFs and the corresponding population VIF constructed from the average (at each time-point) of the individual VIFs after individual temporal adjustments to align peak intensities to the same time-point. **Supplemental Table S4.** Leakage rate, Ki, generated with Patlak model and population averaged vascular input function from internal carotid artery. **Supplemental Table S5.** Leakage rate, Ki, generated with Patlak model and individual vascular input function from sigmoid or transverse vein. **Supplemental Table S6.** Leakage rate, Ki, generated with Patlak model and individual vascular input function from internal carotid artery. **Supplemental Table S7.** Blood volume fraction, vb, generated with Patlak model and population averaged vascular input function from internal carotid artery. **Supplemental Table S8.** Blood volume fraction, vb, generated with Patlak model and individual vascular input function from venous sinus. **Supplemental Table S9.** Blood volume fraction, vb, generated with Patlak model and individual vascular input function from internal carotid artery. **Supplemental Table S10.** Reliablity statistics for the kinetic parameter estimates with four different vascular input functions: individual and population averaged, arterial and venous. **Supplemental Figure S2.** Mean (SD) signal variation plotted separately for the patients and controls, showing similar trends in both groups. **Supplemental Table S11-1.** Correlation between leakage rates, Ki, of the regions and cerebrospinal fluid parameters and symptom duration. Values are Spearman's Rho, 95 % confidence interval (*p* value). **Supplemental Table S11-2.** Correlation between leakage rates, Ki, of the regions and fatigue, clinical composite score and cognitive tests. Values are Spearman's Rho, 95 % confidence interval (*p* value).

## Data Availability

The datasets used and/or analysed during the current study are available from the corresponding author on reasonable request. The data are not publicly available due to privacy or ethical restrictions.

## Abbreviations

3D: Three-dimensional
BBB: Blood-brain barrier
CSF: Cerebrospinal fluid
DCE: Dynamic contrast-enhanced
MRI: Magnetic resonance imaging
VIF: Vascular input function
