# Supplementary material for: Dynamic contrast-enhanced MRI shows altered blood–brain barrier function of deep gray matter structures in neuroborreliosis: a case–control study
Source: Eur Radiol Exp. 2023 Sep 15;7:52. doi: 10.1186/s41747-023-00365-6 (PMC10501980; doi:10.1186/s41747-023-00365-6)
Supplement: Supplementary file 1 — Additional file 1: Supplemental Table S1. Scan parameters for the imaging sequences in the study. Supplemental Table S2. Volumes (mean (standard deviation)) of the selected anatomical tissue masks that were generated by automatic segmentation of the 3D T1 acquisition, and p-values from t-tests for group comparison. Supplemental Table S3. Area under the curve of the signal intensity-to-time plot per region, and p-values from Mann–Whitney U tests for group comparison. Supplemental Figure S1 shows plots of all the individual venous VIFs and the corresponding population VIF constructed from the average (at each time-point) of the individual VIFs after individual temporal adjustments to align peak intensities to the same time-point. Supplemental Table S4. Leakage rate, Ki, generated with Patlak model and population averaged vascular input function from internal carotid artery. Supplemental Table S5. Leakage rate, Ki, generated with Patlak model and individual vascular input function from sigmoid or transverse vein. Supplemental Table S6. Leakage rate, Ki, generated with Patlak model and individual vascular input function from internal carotid artery. Supplemental Table S7. Blood volume fraction, vb, generated with Patlak model and population averaged vascular input function from internal carotid artery. Supplemental Table S8. Blood volume fraction, vb, generated with Patlak model and individual vascular input function from venous sinus. Supplemental Table S9. Blood volume fraction, vb, generated with Patlak model and individual vascular input function from internal carotid artery. Supplemental Table S10. Reliablity statistics for the kinetic parameter estimates with four different vascular input functions: individual and population averaged, arterial and venous. Supplemental Figure S2. Mean (SD) signal variation plotted separately for the patients and controls, showing similar trends in both groups. Supplemental Table S11-1. Correlation between leakage rates, Ki, of the [file 41747_2023_365_MOESM1_ESM.docx]

## **Dynamic contrast-enhanced MRI shows altered blood-brain barrier function of deep grey matter structures in neuroborreliosis: a case-control study**

ELECTRONIC SUPPLEMENTARY MATERIAL

**Supplemental table S1.** Scan parameters for the imaging sequences in the study

| Description | T1 sag mprage | T1 vibe tra flip | DCE vibe-twist |
| --- | --- | --- | --- |
| Voxel size (mm) | 1.0x1.0x1.1 | 1.3x1.3x3.0 | 1.6x1.6x3.0 |
| FOV read (mm) | 256 | 256 | 256 |
| FOV phase | 96.9% | 100% | 100% |
| Phase encoding | A>>P | A>>P | R>>L |
| TR/TE/TI (ms) | 2300/2.98/900 | 4.09/1.39/na | 2.88/0.98/na |
| Flip angle (deg) | 8 | 2.0 and 15.0 | 20.0 |
| Band width (Hz/Px) | 240 | 390 | 1040 |
| Turbo factor | 176 | na | na |
| Averages | 1 | 8 | 1 |
| Temporal resolution (s) | na | na | 7.2 |

DCE dynamic contrast-enhanced; deg degrees; FOV field of view; Hz Herz; mprage magetization-prepared rapid acquisition gradient echo; Px pixel; s seconds; sag sagittal; TE echo time; TI inversion time; TR repetition time; tra transversal; vibe volumetric interpolated breath-hold examination

**Supplemental table S2.** Volumes (mean (standard deviation)) of the selected anatomical tissue masks that were generated by automatic segmentation of the 3D T1 acquisition, and *p*-values from *t*-tests for group comparison.

| Mask volumes (mL) | Patients n=55 | Controls n=15 | *p-*value |
| --- | --- | --- | --- |
| Frontal cortex^a^ | 78.0 (12.4) | 81.6 (11.2) | 0.626 |
| Parietal cortex^a^ | 64.9 (6.5) | 65.6 (9.5) | 0.746 |
| Temporal cortex^a^ | 81.5 (8.9) | 81.8 (10.0) | 0.899 |
| Thalamus | 13.9 (1.5) | 13.5 (1.7) | 0.378 |
| Caudate | 7.4 (0.9) | 6.9 (1.0) | 0.074 |
| Putamen | 9.9 (1.1) | 9.4 (1.1) | 0.157 |
| Hippocampus | 8.3 (1.2) | 8.5 (1.0) | 0.537 |
| Brain stem | 21.9 (2.4) | 21.2 (2.0) | 0.318 |
| Cerebellum white matter | 28.6 (3.6) | 27.8 (2.9) | 0.454 |

^a^Freesurfer label names for the cortical masks used were: frontal - superiorfrontal, caudalmiddlefrontal, rostralmiddlefrontal; parietal - superiorparietal, inferiorparietal, supramarginal; temporal - superiortemporal, middletemporal, inferiortemporal.

**Supplemental table S3.** Area under the curve of the signal intensity-to-time plot per region, and *p*-values from Mann-Whitney *U* tests for group comparison.

| AUC (a.u. x sec) | Patients n=55 mean (SD) | Controls n=15 mean (SD) | Mean difference  (95% CI) | Mann-Whitney *U* test  *p*-value (Bonferroni) |
| --- | --- | --- | --- | --- |
| Frontal cortex | 16.90 (4.44) | 22.37 (15.02) | -5.47  (-13.85 – 2.91) | 0.238 |
| Parietal cortex | 15.45 (4.11) | 17.07 (4.64) | -1.62  (-4.08 – 0.84) | 0.181 |
| Temporal cortex | 20.69 (5.72) | 19.35 (5.38) | 1.34  (1.94 – 4.63) | 0.379 |
| Thalamus | 10.93 (2.134) | 14.09 (3.63) | -3.16  (-5.25 - -1.08) | 0.002* (0.018*) |
| Caudate | 10.34 (2.00) | 13.49 (2.87) | -3.16  (-4.44 - -1.87) | <0.001* (<0.01*) |
| Putamen | 11.537 (2.10) | 11.46 (1.84) | -0.09  (-1.28 – 1.10) | 0.892 |
| Hippocampus | 14.80 (6.48) | 20.37 (5.78) | -5.57  (-9.25 - -1.88) | <0.001* (<0.01*) |
| Brainstem | 12.05 (2.85) | 11.16 (2.79) | 0.89  (-0.76 – 2.54) | 0.181 |
| Cerebellum  white matter | 7.78 (1.63) | 7.26 (1.64) | 0.52  (-0.43 – 1.47) | 0.232 |

a.u. Arbitrary unit; AUC Area under the curve; CI Confidence interval; SD Standard deviation

**Supplemental figure S1** shows plots of all the individual venous VIFs and the corresponding population VIF constructed from the average (at each time-point) of the individual VIFs after individual temporal adjustments to align peak intensities to the same time-point.


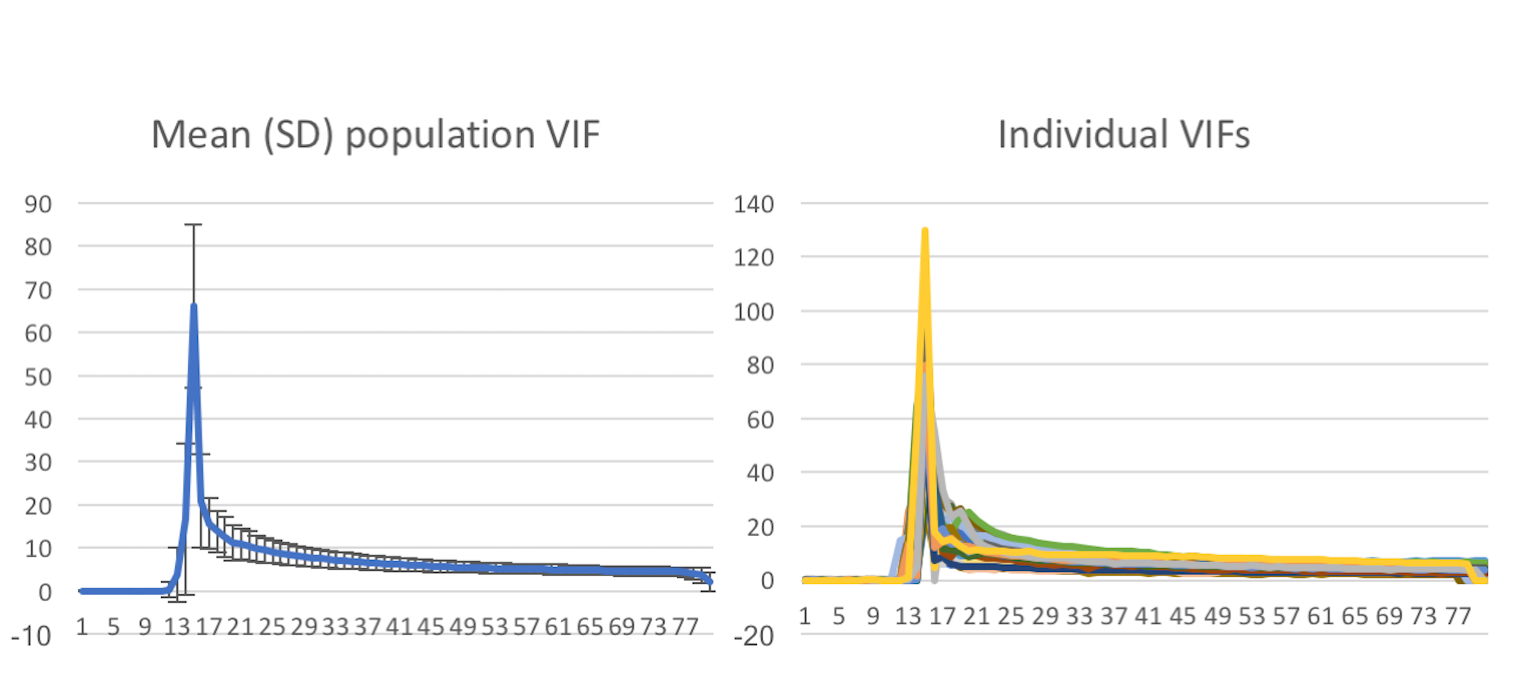


SD Standard deviation; VIF Vascular input function

**Supplemental table S4.** Leakage rate, K_i_, generated with Patlak model and population averaged vascular input function from internal carotid artery.

| K_i_ (x10^-3^/min) | Patients n=55 mean (SD) | Controls n=15 mean (SD) | Mean difference  (95% CI) | Mann-Whitney *U* test  *p*-value (Bonferroni) |
| --- | --- | --- | --- | --- |
| Frontal cortex | 1.42 (0.52) | 2.11 (2.50) | -0.69  (-2.08 – 0.70) | 0.824 |
| Parietal cortex | 0.94 (0.31) | 1.39 (1.09) | -0.44  (-1.05 – 0.16) | 0.046* (0.414) |
| Temporal cortex | 1.75 (0.52) | 1.41 (0.49) | 0.33  (0.04 – 0.63) | 0.007* (0.063) |
| Thalamus | 0.59 (0.10) | 0.75 (0.26) | -0.16  (-0.30 - -0.01) | 0.002* (0.018*) |
| Caudate | 0.56 (0.13) | 0.66 (0.09) | -0.09  (-0.16 - -0.01) | 0.001* (0.009*^)^ |
| Putamen | 0.54 (0.09) | 0.53 (0.06) | 0.01  (-0.04 – 0.06) | 0.506 |
| Hippocampus | 0.87 (0.23) | 1.01 (0.19) | -0.15  (-0.28 - -0.02) | 0.008* (0.072) |
| Brainstem | 0.71 (0.16) | 0.63 (0.09) | 0.09  (0.001 – 0.17 | 0.031* (0.279) |
| Cerebellum  white matter | 0.50 (0.07) | 0.46 (0.06) | 0.04  (-0.001 – 0.08) | 0.049* (0.441) |

CI Confidence interval; SD Standard deviation; K_i_ Leakage rate

**Supplemental table S5.** Leakage rate, K_i_, generated with Patlak model and individual vascular input function from sigmoid or transverse vein.

| K_i_ (x10^-3^/min) | Patients n=55  Mean (SD) | Controls n=15 Mean (SD) | Mean difference  (95% CI) | Mann-Whitney *U* test  *p*-value (Bonferroni) |
| --- | --- | --- | --- | --- |
| Frontal cortex | 1.46 (0.59) | 1.96 (1.90) | -0.50  (-1.56 – 0.56) | 0.758 |
| Parietal cortex | 1.04 (0.60) | 1.37 (0.98) | -0.34  (-0.90 – 0.22) | 0.293 |
| Temporal cortex | 1.81 (0.74) | 1.42 (0.59) | 0.40  (-0.02 – 0.81) | 0.026* (0.234) |
| Thalamus | 0.64 (0.23) | 0.78 (0.29) | -0.14  (-0.28 – 0.01) | 0.080 |
| Caudate | 0.61 (0.23) | 0.70 (0.23) | -0.09  (-0.22 – 0.05) | 0.131 |
| Putamen | 0.57 (0.16) | 0.55 (0.16) | 0.02  (-0.07 – 0.11) | 0.506 |
| Hippocampus | 0.94 (0.39) | 1.10 (0.42) | -0.16  (-0.39 – 0.07) | 0.095 |
| Brainstem | 0.76 (0.19) | 0.64 (0.19) | 0.11  (-0.01 – 0.24) | 0.117 |
| Cerebellum  white matter | 0.52 (0.17) | 0.46 (0.14) | 0.06  (-0.03 – 0.16) | 0.167 |

CI Confidence interval; SD Standard deviation; K_i_ Leakage rate

**Supplemental table S6.** Leakage rate, K_i_, generated with Patlak model and individual vascular input function from internal carotid artery.

| K_i_ (x10^-3^/min) | Patients n=55  Mean (SD) | Controls n=15 Mean (SD) | Mean difference  (95% CI) | Mann-Whitney *U* test  *p*-value (Bonferroni) |
| --- | --- | --- | --- | --- |
| Frontal cortex | 1.22 (0.68) | 1.82 (1.74) | -0.60  (-1.57 – 0.38) | 0.138 |
| Parietal cortex | 0.80 (0.48) | 1.19 (0.59) | -0.40  (-0.69 – -0.10) | 0.001* (0.009*) |
| Temporal cortex | 1.38 (0.58) | 1.30 (0.39) | 0.08  (-0.23 – 0.40) | 0.937 |
| Thalamus | 0.49 (0.19) | 0.70 (0.19) | -0.21  (-0.32 - -0.10) | 0.001* (0.009*) |
| Caudate | 0.48 (0.23) | 0.66 (0.20) | -0.18  (-0.30 - -0.05) | 0.002* (0.018*) |
| Putamen | 0.45 (0.17) | 0.51 (0.15) | -0.06  (-0.16 – 0.03) | 0.159 |
| Hippocampus | 0.76 (0.37) | 1.04 (0.37) | -0.28  (-0.49 - -0.07) | 0.004* (0.036*) |
| Brainstem | 0.60 (0.27) | 0.61 (0.27) | 0.01  (-0.16 – 0.15) | 0.683 |
| Cerebellum  white matter | 0.38 (0.15) | 0.39 (0.11) | 0.01  (-0.09 – 0.08) | 0.515 |

CI Confidence interval; SD Standard deviation; K_i_ Leakage rate

**Supplemental table S7.** Blood volume fraction, v_b_, generated with Patlak model and population averaged vascular input function from internal carotid artery.

| v_b_ | Patients n=55  Mean (SD) | Controls n=15 Mean (SD) | Mean difference  (95% CI) | Mann-Whitney *U* test  *p*-value (Bonferroni) |
| --- | --- | --- | --- | --- |
| Frontal cortex | 0.54 (0.14) | 0.67 (0.39) | -0.13  (-0.35 – 0.09) | 0.334 |
| Parietal cortex | 0.51 (0.12) | 0.58 (0.22) | -0.07  (-0.16 – 0.01) | 0.274 |
| Temporal cortex | 0.64 (0.17) | 0.61 (0.17) | 0.03  (-0.07 – 0.13) | 0.379 |
| Thalamus | 0.38 (0.08) | 0.48 (0.12) | -0.10  (-0.17 - -0.03) | 0.003* (0.027*) |
| Caudate | 0.35 (0.08) | 0.46 (0.09) | -0.11  (-0.15 - -0.06) | 0.001* (0.009*) |
| Putamen | 0.40 (0.07) | 0.40 (0.07) | 0.01  (-0.04 – 0.04) | 0.994 |
| Hippocampus | 0.49 (0.24) | 0.65 (0.19) | -0.16  (-0.30 - -0.02) | 0.001* (0.009*) |
| Brainstem | 0.43 (0.12) | 0.40 (0.12) | 0.03  (-0.04 – 0.10) | 0.154 |
| Cerebellum  white matter | 0.28 (0.06) | 0.26 (0.06) | 0.02  (-0.02 – 0.05) | 0.293 |

CI Confidence interval; SD Standard deviation; v_b_ Blood volume fraction

**Supplemental table S8.** Blood volume fraction, v_b_, generated with Patlak model and individual vascular input function from venous sinus.

| v_b_ | Patients n=55 Mean (SD) | Controls n=15 Mean (SD) | Mean difference  (95% CI) | Mann-Whitney *U* test  *p*-value (Bonferroni) |
| --- | --- | --- | --- | --- |
| Frontal cortex | 0.48 (0.2) | 0.50 (0.30) | -0.02  (-0.15 – 0.11) | 0.847 |
| Parietal cortex | 0.46 (0.25) | 0.46 (0.24) | 0.01  (-0.14 – 0.15) | 0.881 |
| Temporal cortex | 0.59 (0.36) | 0.49 (0.23) | 0.10  (-0.10 – 0.29) | 0.232 |
| Thalamus | 0.34 (0.16) | 0.38 (0.13) | -0.04  (-0.13 – 0.05) | 0.186 |
| Caudate | 0.32 (0.14) | 0.37 (0.12) | -0.04  (-0.12 – 0.04) | 0.101 |
| Putamen | 0.35 (0.13) | 0.32 (0.09) | 0.04  (-0.03 – 0.11) | 0.562 |
| Hippocampus | 0.47 (0.53) | 0.51 (0.19) | -0.05  (-0.33 – 0.23) | 0.047* (0.423) |
| Brainstem | 0.39 (0.23) | 0.32 (0.15) | 0.07  (-0.06 – 0.19) | 0.210 |
| Cerebellum  white matter | 0.25 (0.14) | 0.21 (0.08) | 0.04  (-0.03 – 0.12) | 0.341 |

CI Confidence interval; SD Standard deviation; v_b_ Blood volume fraction

**Supplemental table S9.** Blood volume fraction, v_b_, generated with Patlak model and individual vascular input function from internal carotid artery.

| v_b_ | Patients n=55  Mean (SD) | Controls n=15 Mean (SD) | Mean difference  (95% CI) | Mann-Whitney *U* test  *p*-value (Bonferroni) |
| --- | --- | --- | --- | --- |
| Frontal cortex | 0.49 (0.15) | 0.68 (0.63) | -0.19  (-0.54 – 0.16) | 0.534 |
| Parietal cortex | 0.46 (0.13) | 0.59 (0.33) | -0.13  (-0.32 – 0.05) | 0.334 |
| Temporal cortex | 0.58 (0.18) | 0.61 (0.24) | -0.04  (-0.18 – 0.11) | 0.937 |
| Thalamus | 0.35 (0.10) | 0.48 (0.18) | -0.13  (-0.24 - -0.03) | 0.005* (0.045*) |
| Caudate | 0.35 (0.09) | 0.48 (0.16) | -0.13  (-0.22 - -0.04) | 0.003* (0.027*) |
| Putamen | 0.39 (0.11) | 0.42 (0.16) | 0.03  (-0.10 – 0.04) | 0.769 |
| Hippocampus | 0.44 (0.20) | 0.65 (0.25) | -0.21  (-0.33 - -0.08) | 0.002* (0.018*) |
| Brainstem | 0.39 (0.12) | 0.40 (0.15) | 0.01  (-0.08 – 0.07) | 0.983 |
| Cerebellum  white matter | 0.26 (0.07) | 0.26 (0.09) | 0.002  (-0.05 – 0.04) | 0.960 |

CI Confidence interval; SD Standard deviation; v_b_ Blood volume fraction

***Vascular input function and kinetic parameter estimates – agreement statistics***

The agreement or reliability of measurement (or estimate) of kinetic parameters with application of the various types of vascular input function was assessed. Calculation of intraclass correlation coefficient (ICC) was based on the following form: single measurement, absolute agreement and 2-way mixed effects model [1]. In the table, Cronbach’s alpha is also provided as this is a measure of consistency or correlation between the items, rather than absolute agreement. There are only three out of 18 estimates with Cronbach’s alpha value below 0.70 and the remaining range from 0.70-0.97, and we consider the overall consistency between the various vascular input functions for kinetic parameter estimates to be good [2]. In terms of absolute agreement – which may be somewhat artificial and strict in the setting of these various vascular input functions - the intraclass correlation coefficients range from 0.14-0.89 with 10/18 values below 0.5 (poor reliability), 6/18 in the range 0.50-0.75 (moderate reliability) and 2/18 in the range 0.75-0.9 (good reliability).

**Supplemental table S10.** Reliablity statistics for the kinetic parameter estimates with four different vascular input functions: individual and population averaged, arterial and venous.

|  | Leakage rate (K_i_) | | Blood volume fraction (v_b_) | |
| --- | --- | --- | --- | --- |
|  | Cronbach’s α | ICC (95% CI) | Cronbach’s α | ICC (95% CI) |
| Frontal cortex | 0.97 | 0.89 (0.85-0.93) | 0.84 | 0.55 (0.44-0.66) |
| Parietal cortex | 0.94 | 0.78 (0.69-0.85) | 0.79 | 0.48 (0.34-0.60) |
| Temporal cortex | 0.86 | 0.57 (0.44-0.69) | 0.79 | 0.47 (0.35-0.60) |
| Thalamus | 0.79 | 0.45 (0.32-0.58) | 0.75 | 0.41 (0.29-0.54) |
| Caudate | 0.82 | 0.51 (0.39-0.63) | 0.70 | 0.35 (0.23-0.48) |
| Putamen | 0.41 | 0.14 (0.04-0.26) | 0.62 | 0.26 (0.14-0.39) |
| Hippocampus | 0.82 | 0.52 (0.40-0.64) | 0.87 | 0.62 (0.52-0.72) |
| Brain stem | 0.81 | 0.49 (0.36-0.61) | 0.81 | 0.50 (0.38-0.62) |
| Cerebellum WM | 0.67 | 0.28 (0.15-0.42) | 0.77 | 0.43 (0.31-0.56) |

CI Confidence interval; ICC Intraclass correlation coefficient; WM White matter

***Signal drift***

To assess any systematic temporal signal drift during the dynamic imaging, an identical DCE sequence without contrast agent administration was acquired in 14 subjects (ten controls and four patients). These scans were conducted between December 13th, 2017 and July 10th, 2018. Intra-axial parenchymal voxels were identified (by thresholding) from a single central slice in each subject, and the relative time-dependent change in DCE signal intensity was measured. Temporal signal drift from the non-enhanced DCE series was analyzed by linear regression and analysis of variance (ANOVA) across all subjects. Separate regression analysis was also performed for the controls and patients for visual inspection of any group differences.

The signal drift analysis revealed a small, but significant, linear reduction in signal intensity over the time-course of the DCE series. The slope of the linear regression was -0.002 (R^2^=0.09, *p*<0.01, ANOVA F=110.4, *p*<0.01). The relative change was 0.14-0.16 % negative scanner drift per minute, and this is comparable to other groups that report 0.08 – 0.20 % scanner drift per minute [3, 4]. This negative drift may have caused a slight under-estimation of leakage rates in both cohorts, but would be expected to have the same effect in both groups.

**Supplemental figure S2.** Mean (SD) signal variation plotted separately for the patients and controls, showing similar trends in both groups.


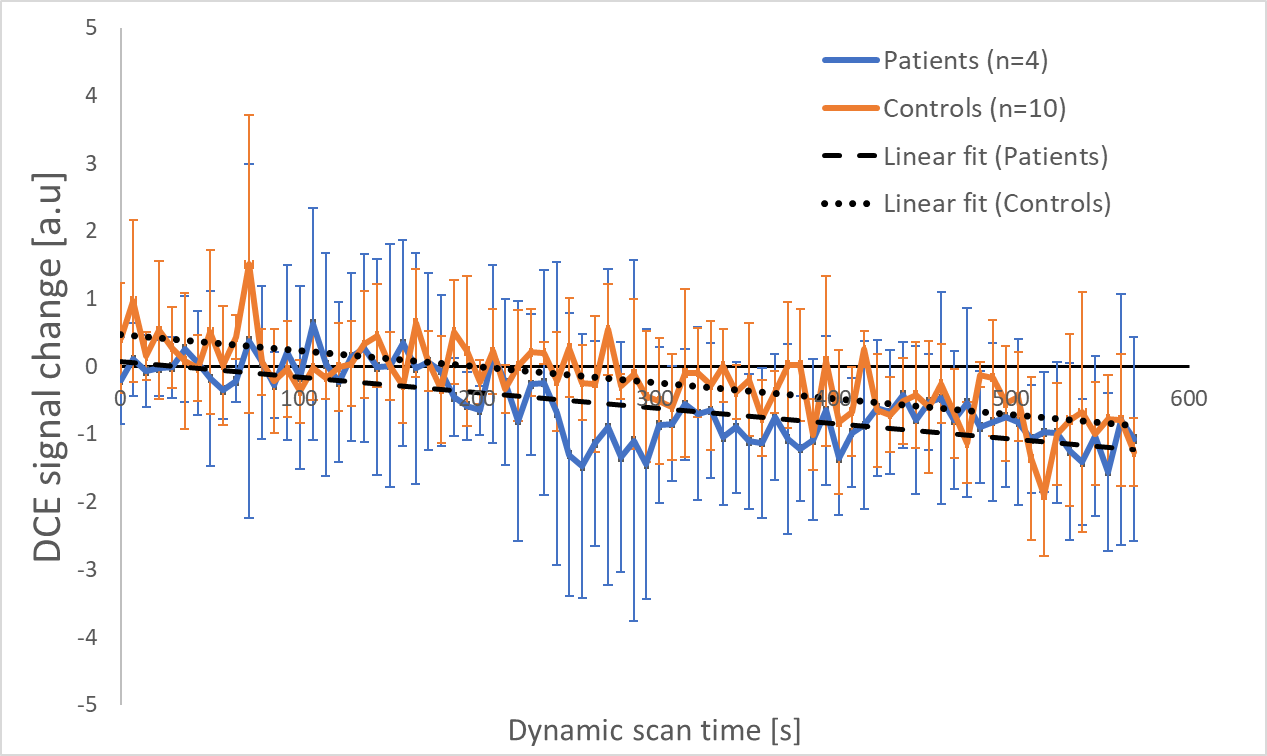


Although signal drift was assessed, it was only performed within a relatively limited space of time compared to the overall duration of patient inclusion. Signal drift may have been different in the first two years of patient inclusion when no control subjects were scanned.

***Correlation analysis***

**Supplemental table S11-1.** Correlation between leakage rates, K_i_, of the regions and cerebrospinal fluid parameters and symptom duration. Values are Spearman’s Rho, 95 % confidence interval (*p* value).

| K_i_ (min^-1^) | CSF-serum albumin ratio  (N=52) | CSF protein  (N=54) | CSF cells/mm^3^  (N=55) | Days CSF - MRI  (N=55) | Symptom duration  (N=49) |
| --- | --- | --- | --- | --- | --- |
| Frontal cortex | -0.19, -0.45-0.09  (0.169) | -0.10, -0.36-0.17  (0.476) | 0.08, -0.19-0.34  (0.577) | 0.02, -0.25-0.28  (0.884) | 0.06, -0.22-0.34  (0.669) |
| Parietal cortex | -0.04, -0.31-0.24  (0.801) | -0.02, -0.29-0.25  (0.883) | 0.20, -0.08-0.44  (0.154) | 0.09, -0.18-0.35  (0.511) | 0.07, -0.22-0.34  (0.647) |
| Temporal cortex | 0.10, -0.18-0.36  (0.490) | 0.07, -0.20-0.34  (0.595) | -0.08, -0.33-0.20  (0.588) | -0.10, -0.35-0.17  (0.484) | -0.01, -0.29-0.27  (0.925) |
| Thalamus | 0.06, -0.22-0.33  (0.684) | -0.04, -0.31-0.23  (0.755) | 0.14, -0.13-0.40  (0.302) | -0.04, -0.30-0.23  (0.788) | -0.08, -0.36-0.20  (0.567) |
| Caudate | 0.13, -0.15-0.39  (0.373) | -0.01, -0.28-0.26  (0.956) | 0.10, -0.17-0.36  (0.451) | -0.13, -0.38-0.14  (0.354) | -0.02, -0.30-0.26  (0.885) |
| Putamen | 0.02, -0.26-0.29  (0.902) | -0.07, -0.33-0.20  (0.609) | -0.13, -0.38-0.15  (0.358) | -0.10, -0.36-0.17  (0.475) | -0.09, -0.37-0.19  (0.524) |
| Hippocampus | -0.31, -0.54- -0.03 (0.026*/0.234^a^) | -0.39, -0.60- -0.12 (0.004*/0.036*^a)^ | - 0.13, -0.38-0.14  (0.338) | 0.07, -0.20-0.33  (0.599) | -0.26, -0.51-0.03  (0.074) |
| Brainstem | 0.18, -0.10-0.43  (0.200) | 0.13, -0.14-0.39  (0.339) | 0.14, -0.13-0.39  (0.312) | 0.003, -0.26-0.27  (0.984) | 0.03, -0.26-0.30  (0.866) |
| Cerebellum  white matter | 0.23, -0.05-0.48  (0.097) | 0.18, -0.09-0.43  (0.190) | 0.19, -0.08-0.44  (0.165) | -0.07, -0.33-0.20  (0.625) | -0.09, -0.36-0.20  (0.539) |

^a^adjusted p value (Bonferroni correction)

**Supplemental table S11-2.** Correlation between leakage rates, K_i_, of the regions and fatigue, clinical composite score and cognitive tests. Values are Spearman’s Rho, 95 % confidence interval (*p* value).

| K_i_ (min^-1^) | FSS  early phase  (N=52) | FSS  six months  (N=46) | Clinical score early phase  (N=47) | Clinical score  six months  (N=43) | Digit span-F  early phase  (N=55) | Digit span-B early phase  (N=55) | Verbal memory  six months  (N=42) |
| --- | --- | --- | --- | --- | --- | --- | --- |
| Frontal cortex | -0.09,  -0.36-0.20  (0.544) | -0.01,  -0.31-0.29  (0.943) | -0.06,  -0.34–0.23  (0.705) | -0.13,  -0.42-0.19  (0.409) | 0.06,  -0.22-0.33  (0.665) | 0.08,  -0.20-0.34  (0.571) | 0.28,  -0.03-0.55  (0.069) |
| Parietal cortex | 0.24,  -0.05-0.49  (0.089) | 0.16,  -0.14-0.44  (0.279) | 0.23,  -0.27-0.31  (0.878) | 0.13,  -0.19-0.42  (0.421) | 0.01,  -0.27-0.28  (0.967) | 0.01,  -0.26-0.29  (0.917) | 0.20,  -0.12-0.48  (0.202) |
| Temporal cortex | 0.17,  -0.12-0.43  (0.243) | 0.15,  -0.16-0.43  (0.329) | -0.15,  -0.42-0.14  (0.303) | 0.03,  -0.28-0.34  (0.849) | -0.14,  -0.40-0.13  (0.293) | -0.07,  -0.33-0.21  (0.629) | 0.13,  -0.19-0.42  (0.424) |
| Thalamus | -0.01,  -0.29-0.27  (0.935) | -0.10,  -0.39-0.20  (0.493) | 0.05,  -0.24-0.33  (0.742) | 0.15,  -0.17-0.44  (0.345) | 0.20,  -0.08-0.45  (0.141) | 0.20,  -0.08-0.45  (0.144) | 0.26,  -0.06, 0.53  (0.098) |
| Caudate | -0.05,  -0.32-0.24  (0.745) | -0.06,  -0.36-0.24  (0.677) | -0.11,  -0.39-0.18  (0.455) | 0.20,  -0.12-0.48  (0.200) | 0.15,  -0.12-0.41  (0.261) | 0.04,  -0.23-0.31  (0.754) | 0.13,  -0.19-0.43  (0.407) |
| Putamen | 0.06,  -0.22-0.34  (0.659) | -0.06,  -0.35-0.25  (0.705) | -0.07,  -0.35-0.23  (0.658) | 0.07,  -0.24-0.37  (0.652) | 0.07,  -0.21-0.33  (0.627) | -0.04  -0.31-0.24  (0.787) | 0.21,  -0.11-0.49  (0.179) |
| Hippocampus | 0.04,  -0.24-0.32  (0.777) | 0.08,  -0.22-0.37  (0.583) | -0.14,  -0.41-0.16  (0.360) | 0.11,  -0,20-0.41  (0.470) | 0.09,  -0.19-0.35  (0.516) | 0.13,  -0.15-0.39  (0.342) | -0.09,  -0.40-0.23  (0.552) |
| Brainstem | 0.13,  -0.16-0.39  (0.367) | -0.03,  -0.33-0.27  (0.825) | -0.01,  -0.30-0.28  (0.928) | 0.09,  -0.23-0.39  (0.570) | -0.14,  -0.40-0.14  (0.309) | -0.09,  -0.35-0.19  (0.532) | 0.03,  -0.28-0.34  (0.847) |
| Cerebellum  white matter | 0.10,  -0.18-0.37  (0.464) | 0.12,  -0.19-0.40  (0.439) | 0.09,  -0.21-0.37  (0.566) | 0.24,  -0.07-0.51  (0.119) | -0.09,  -0.35-0.19  (0.525) | -0.08,  -0.35-0.20  (0.565) | 0.15,  -0.17-0.44  (0.344) |

***References:***

1. Koo TK, Li MY (2016) A Guideline of selecting and reporting Intraclass Correlation Coefficients for reliability research. J Chiropr Med 15:155—163. <https://doi.org/10.1016/j.jcm.2016.02.012>

2. Tavakol M, Dennick R (2011) Making sense of Cronbach's alpha. Int J Med Educ 2:53—55. <https://doi.org/10.5116/ijme.4dfb.8dfd>

3. Heye AK, Thrippleton MJ, Armitage PA, et al. (2016) Tracer kinetic modelling for DCE-MRI quantification of subtle blood-brain barrier permeability. Neuroimage 125:446—455. <https://doi.org/10.1016/j.neuroimage.2015.10.018>

4. Varatharaj A, Liljeroth M, Darekar A, Larsson HBW, Galea I, Cramer SP (2019) Blood-brain barrier permeability measured using dynamic contrast-enhanced magnetic resonance imaging: a validation study. J Physiol 597:699—709. <https://doi.org/10.1113/JP276887>
